# The Physiologic Basis of Molecular Therapeutics for Peripheral Nerve Injury: A Primer

**DOI:** 10.1016/j.jhsg.2024.01.017

**Published:** 2024-03-26

**Authors:** Marie C. Spezia, Christopher J. Dy, David M. Brogan

**Affiliations:** ∗ University of Missouri-Columbia School of Medicine, Columbia, MO; † The Institute of Clinical and Translational Sciences and Clinical Research Training Center, Washington University, St. Louis, MO; ‡ Department of Orthopaedic Surgery, Washington University School of Medicine, St. Louis, MO

**Keywords:** Neurotrophic factors, Nerve regeneration, Peripheral nerve injury, PEG fusion, Tacrolimus

## Abstract

Peripheral nerve injuries affect a significant number of patients who experience trauma affecting the hand and upper extremity. Improving unsatisfactory outcomes from repair of these injuries remains a clinical challenge despite advancements in microsurgical repair. Imperfections of the nerve regeneration process, including imprecise reinnervation, distal axon degradation, and muscular atrophy, complicate the repair process. However, the capacity for peripheral nerves to regenerate offers an avenue for therapeutic advancement. Regeneration is a temporally and spatially dynamic process coordinated by Schwann cells and neurons among other cell types. Neurotrophic factors are a primary means of controlling cell growth and differentiation in the repair setting. Sustained axon survival and regrowth and consequently functional outcomes of nerve repair in animal models are improved by the administration of neurotrophic factors, including glial cell-derived neurotrophic factor, nerve growth factor, sterile alpha and TIR motif containing 1, and erythropoietin. Targeted and sustained delivery of neurotrophic factors through gelatin-based nerve conduits, multiluminal conduits, and hydrogels have been shown to enhance the innate roles of these factors to promote expedient and accurate peripheral nerve regeneration in animal models. These delivery methods may help address the practical limitations to clinical use of neurotrophic factors, including systemic side effects and the need for carefully timed, precisely localized release schedules. In addition, tacrolimus has also improved peripheral nerve regrowth in animal models and has recently shown promise in addressing human disease. Ultimately, this realm of adjunct pharmacotherapies provides ample promise to improve patient outcomes and advance the field of peripheral nerve repair.

Advancement in pharmacotherapeutics for hand and upper extremity peripheral nerve injury is critically needed. Approximately 2.3% of all patients with trauma to an extremity experience some form of peripheral nerve injury.^1^ Especially in the realm of brachial plexus injuries and other peripheral nerve injuries nearer the ventral root, the slow rate of axonal regeneration can lead to poor outcomes even after prompt and technically proficient microsurgical nerve repair.^2^, ^3^, ^4^ Both the degeneration of motor neurons, which usually occurs within 2 weeks, and the atrophy of target muscles in the absence of active reinnervation can result in detrimental and permanent functional loss.^5^^,^^6^ The hallmark difficulties of peripheral nerve repair can be thought of through the lens of three overarching tenants: a struggle to bridge gaps, a tendency for axonal growth along stray paths, and a failure to reconnect vital supply routes between the cell body and distal segment before axonal degeneration and muscle atrophy take hold, thwarting further recovery. Despite improvements in our understanding of nerve pathophysiology and advancements in surgical techniques, persistently poor clinical outcomes after nerve injury create an opportunity to capitalize on the regenerative process using adjunctive pharmacotherapies and molecular treatments. This review will focus on the role of neurotrophic factors and their application as novel therapeutics to promote axonal survival and growth, thereby improving clinical outcomes following peripheral nerve injuries.

## Schwann Cell and Nerve Regeneration

**Figure 1:**
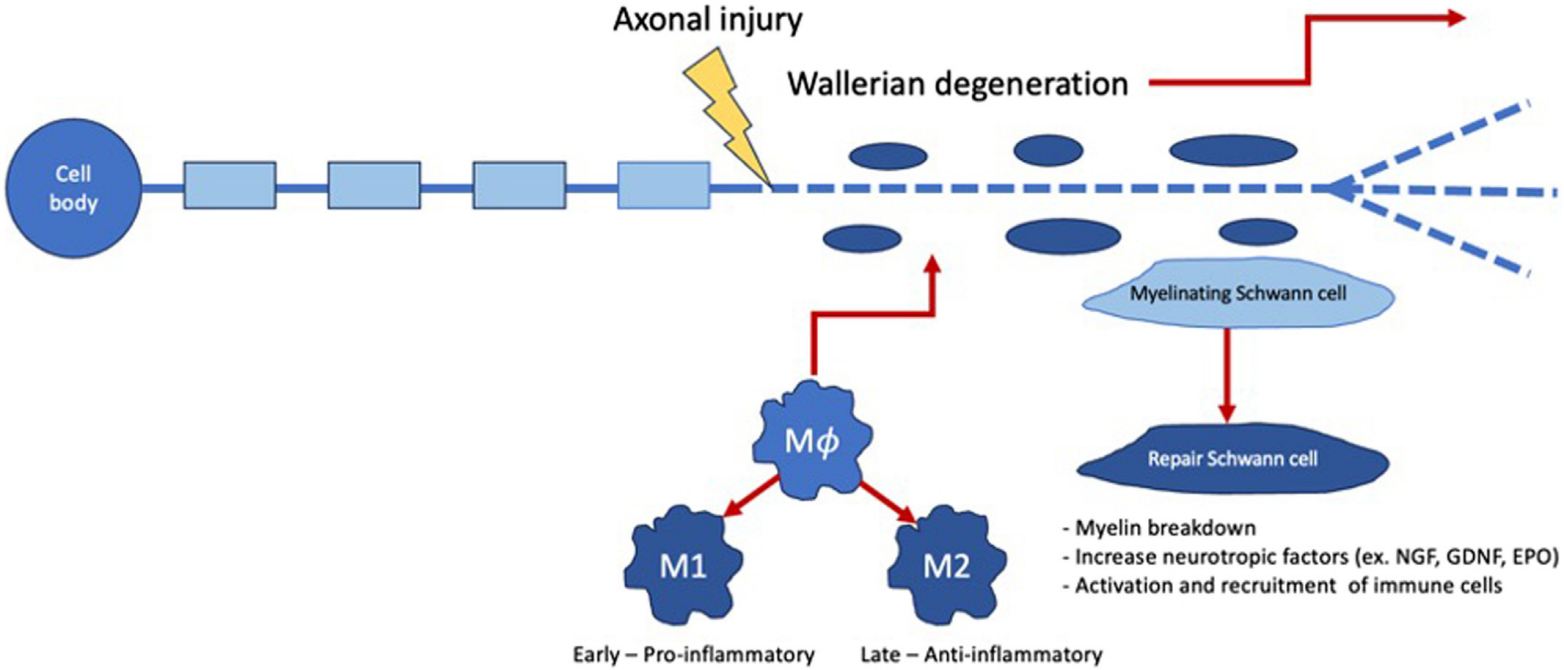
Overview of changes in the cell microenvironment following peripheral nerve damage. Following injury to a peripheral nerve, the segment of the axon distal to the insult and a brief portion proximal to it begins to undergo Wallerian degeneration, an innate immune response dictated by Schwann cells, fibroblasts, macrophages, and endothelial cells.^7^ The molecules produce by these cells drive macrophages to differentiate into 2 subtypes, M1 and M2, which exist in a finely tuned ratio, and Schwann cells to dedifferentiate from a myelinating phenotype into a repair phenotype.^10^^,^^11^ M1 cells are largely proinflammatory and play a major role in myelin clearance from the damaged and degenerating axon, whereas M2 cells are primarily anti-inflammatory and produce neurotrophic factors to create an environment favorable to axon regrowth.^7^^,^^9^ Redifferentiated repair SCs scavenge myelin as well as support and direct the regrowth of new axons toward the target tissue by releasing molecules like NGF into the microenvironment and maintaining a structural scaffolding.^7^^,^^9^

Unlike the central nervous system, peripheral nerves possess the ability to convert to a regenerative state after injury.^7^ Conversion to a regenerative state is inducted by a calcium wave that aids in membrane resealing, growth cone formation, epigenetic alterations, protein synthesis, and production of regenerative signaling factors.^8^, ^9^, ^10^, ^11^ For regeneration to occur, an injured nerve must first go through the process of Wallerian degeneration, a complex innate immune response orchestrated by Schwann cells (SCs), fibroblasts, and macrophages, among others, to clear inhibitory myelin surrounding the axon distal to the site of injury and upregulate neurotrophic factor production (Fig. 1).^12^

Schwann cells are the major glial cell of the peripheral nervous system, a fact underscored by their central role in coordination of peripheral nerve regeneration. Following traumatic nerve injury, denervated SCs switch their phenotype from one dedicated to axonal myelination to one of regenerative capacity within the first 24 hours following injury.^8^ In doing so, SCs must first dedifferentiate from the myelinating phenotype and then redifferentiate to the repair phenotype through a series of biochemical and morphological changes that are dependent on the appropriate timing and intensity of neurotrophic factor release among other signals.^13^ As part of this repair-focused phenotype, SCs induce demyelination of the damaged nerve and upregulate the expression of genes that promote and guide axonal growth and slow neuronal cell death.^14^ These proregenerative SCs also secret extracellular matrix (ECM) packed with progrowth neurotrophic factors along with cytokines and chemokines to recruit immune cells that also play a role in the regenerative process.^12^ Over time, however, growth inhibitory proteins will accumulate in the distal nerve segment, and SCs will lose their regenerative properties.^15^, ^16^, ^17^ The ability to keep SCs in the repair phenotype sits at the center of therapeutic endeavors to promote the continued production of neurotrophic factors to improve axonal survival and directed outgrowth.^18^ Key to this endeavor would be promoting a proregenerative phenotype across all denervated SCs equally, which makes viral vectors, namely adenoviral vectors, a promising future mode of administration of neurotrophic factors as a complement to surgical repair.^19^

## Neurotrophic Factors Involved in Neural Regeneration

Neurotrophic factors are proteins released from SCs and other neuron-associated cells, like fibroblasts and macrophages, in the aftermath of peripheral nerve injury to promote neuron survival and function as well as drive the appropriate differentiation of SCs and macrophages.^20^^,^^21^ Numerous specific neurotrophic factors or regulators have garnered attention as potential therapeutic targets in nerve regeneration. The more recent players on the preclinical stage include glial cell-derived neurotrophic factor (GDNF), nerve growth factor (NGF), sterile alpha and TIR motif containing 1 (SARM1), and erythropoietin (EPO) among others.^22^, ^23^, ^24^, ^25^, ^26^, ^27^, ^28^, ^29^

In terms of therapeutic implications for neurotrophic factors, it should be noted that most factors demonstrate diffuse tissue penetration resulting in off target effects, and a majority of factors have short half-lives, limiting their effectiveness through topical or systemic routes of administration.^30^ To combat their temporal restrictions, preclinical models have employed the introduction of neurotrophic factors using drug-inducible gene therapy. This technique allows for fine-tuned control of expression and minimizes untoward side effects in the nontargeted administration of neurotrophic factors, such as axon entrapment.^30^^,^^31^ Another method of ensuring local delivery is through the use of nerve conduits impregnated with various neurotrophic factors.^32^^,^^33^

Nerve growth factor is one of the best characterized of the neurotrophic factors. NGF is upregulated in two phases at the site of injury and distal to it, resulting in the promotion of axonal survival and growth, particularly of sympathetic and sensory neurons.^10^, ^34^, ^35^ In animal models, NGF has been successfully administered via nerve conduits and fibrin glue membranes to improve peripheral nerve growth and recovery, the localized delivery of which also avoids the negative side effects of systemic NGF administration.^25^^,^^36^, ^37^, ^38^, ^39^ As reviewed by Alastra et al,^40^ preliminary human studies on the subcutaneous or intracerebroventricular administration of NGF to either healthy subjects or patients with peripheral neuropathy produced hyperalgesia at the injection site as well as mild to moderate-severe transient muscle pain in some cases.Click or tap here to enter text. These findings emphasize the need for a localized, timed administration of NGF to minimize side effects. Like many neurotrophic factors, NGF has a short half-life, making the timing and sustained concentration of its delivery challenging. A potential solution to this is the development of unique biocompatible, biodegradable hydrogels to deliver factors like NGF within nerve conduits.^38^^,^^39^

A related but distinct neurotrophic factor is GDNF, belonging to the well-known family of neurotrophins that also includes NGF, brain-derived nerve growth factor (BDNF), and neurotrophins (NT3, NT4/5). GDNF is upregulated by the proregenerative phenotype of SCs and functions specifically to promote motor neuron survival and regeneration as well as myelination enhancement and neuromuscular junction remodeling following injury.^41^, ^42^, ^43^, ^44^, ^45^ Even with the limitations of a short half-life and suboptimal tissue penetration, exogenously administered GDNF has been shown to improve motor neuron survival and functional recovery in vivo.^41^^,^^42^^,^^46^^,^^47^ Skeletal muscle can also produce GDNF to promote neuron survival and thus prevent target muscle atrophy following nerve injury.^44^^,^^48^, ^49^, ^50^ In line with these findings and indicating another potential therapeutic avenue involving GDNF, multiple studies suggest that exercise stimulates the release of GDNF from skeletal muscle, thus upregulating neuronal prosurvival genes.^51^, ^52^, ^53^ Possible therapeutic uses of GDNF are complicated by the intricate timing and localization required for this factor to promote growth; for example, sustained, high local levels of GDNF at the injury site actually induces a coiling formation of new axons and inhibits regeneration by causing entrapment and hypertrophy.^23^^,^^46^^,^^54^, ^55^, ^56^, ^57^ However, carefully timed delivery of GDNF promotes long-term motor neuron survival, stimulates distal axon regeneration, and decreases target muscle atrophy in rat nerve injury models.^30^^,^^54^^,^^56^ Biocompatible nerve conduits integrating GDNF have also shown success in promoting the survival of both neurons and SCs.^58^, ^59^, ^60^ One study found that a novel silk fibroin-based nerve conduit with NGF and GDNF better maintained retrograde transport and provided more neuroprotection compared with autografts and plain silk fibroin conduit.^32^^,^^33^

Recent discoveries of the role of SARM1 as a central executioner of Wallerian degeneration has sparked a flurry of interest in its potential as a therapeutic target.^61^ SARM1 possesses both nicotinamide adenine dinucleotide glycohydrolase and nicotinamide adenine dinucleotide phosphate phosphatase enzyme activities, the former of which has acquired more attention for its role in nicotinamide adenine dinucleotide (NAD) depletion in injured axons as a major contributing factor to Wallerian degeneration by speeding up the depletion of these essential metabolic factors.^62^^,^^63^ The loss of NAD results in the loss of ATP, whereas a lack of NADPH results in the damaging accumulation of reactive oxygen species.^63^^,^^64^ NMNAT2 is the negative regulator of SARM1 preventing the accelerated breakdown of NAD and the accumulation of its own prodegenerative substrate, NMN.^64^, ^65^, ^66^, ^67^ It has been shown in animal models that SARM1 is the central executioner of Wallerian degeneration and that its knockout or knockdown slows neuron degeneration in the cases of nerve injury or degenerative disease.^27^ Although knocking out SARM1 does not entirely prevent axon degeneration, its inhibition may extend the therapeutic window for intervention before degeneration and target muscle atrophy sets in.^68^, ^69^, ^70^ Small molecule inhibitors are currently under development to inhibit SARM1 and potentially slow the degradation of axons following peripheral nerve injury.^71^

Erythropoietin presents an achievable clinical intervention as it has already been proven to be a safe and well-tolerated treatment for anemia since its approval by the United States Food and Drug Administration in 1989.^72^ Systemic EPO therapy’s neuroprotective properties have been shown by its ability to improve functional and electrophysiological outcomes in animal models for neuropathic pain and neuropathies.^73^^,^^74^ Multiple promising studies have shown intraperitoneal EPO injections result in increased axon diameter, myelin thickness, and total number of nerve fibers along with improved motor function outcomes after nerve injuries in animal models.^11^, ^75^, ^76^, ^77^, ^78^, ^79^ Axonal injury stimulates localized EPO production by SCs, which is then thought to bind to EPO-receptors (EPO-R) that are upregulated on adjacent neurons and SCs.^29^^,^^80^ In addition to promoting SC recruitment and migration to the site of injury, the binding of EPO to EPO-R on SCs activates intracellular signaling pathways. These pathways regulate dedifferentiation and proliferation of SCs, inhibition of neuron death by inactivation of apoptotic mediators, and promotion of immune modulatory and antioxidative responses.^29^^,^^81^ More recently and in the context of rat sciatic nerve crush injuries, EPO has been demonstrated to promote the M2 macrophage phenotype. M2 macrophages phagocytose dying SCs and myelin debris from the nerve injury site and attenuate apoptosis of nerves, resulting in improved functional outcomes.^82^ EPO can significantly tamp down the expression of proinflammatory genes while upregulating the expression of anti-inflammatory genes, conferring a neuroprotective environment.^82^

## Tacrolimus as a Neuroregenerative Agent

The macrolide tacrolimus (FK506) may be better known for its use as an immunomodulator with neuropathic side effects; however, this drug has been demonstrated to have a separate neurogenerative function mediated through the FK506 binding protein.^83^^,^^84^ Specifically, tacrolimus has been shown to enhance peripheral nerve regeneration.^85^^,^^86^ When administered in the aftermath of nerve injury, tacrolimus is taken up by axons and forms heterocomplexes with a series of other proteins that are redistributed to the growth cones of injured neurons, resulting in accelerated regeneration.^87^ In animal models, systemic and local administration of tacrolimus improved motor functional outcomes and accelerated axon regeneration.^88^^,^^89^ However, the side effects associated with long-term tacrolimus use, especially nephrotoxicity, have been well established, leading researchers to explore more localized mechanisms of administration. Strategies to date have focused on biocompatible, dose-regulating modalities with hydrogels and microfilms in nerve wraps.^90^, ^91^, ^92^, ^93^

## Avenues of Therapeutic Delivery

Systemic administration of neurotrophic factors comes with a myriad of unwanted side effects and, for reasons discussed above, is incapable of delivering sufficient concentrations to damaged nerves.^36^ Neurotrophic factor production is upregulated in SCs and nerve cells at injury sites, but these endogenous levels are not enough to produce the prosurvival, progrowth microenvironment needed for satisfactory recovery. Clearly, a strategy for better delivery of localized, sustained exogenous administration of one or, preferably, multiple factors is needed.^94^, ^95^, ^96^ Encapsulating factors into gelatin-based nerve conduits or multiluminal conduits as well as integrating factors into hydrogels injected into traditional conduits have all been shown to improve the accuracy and expediency of peripheral nerve regeneration.^24^^,^^39^^,^^96^^,^^97^ In addition to expected benefits, like protecting neurotrophic factors from breakdown, these avenues of delivery allow for the localization of factors at the site of injury and the controlled, long-term release of factors based on the predictable degradation of the substrate material.

## Conclusions

Although preliminary data are generally very promising, each of the above treatment modalities presents its own array of challenges to efficient, effective clinical applications. Future research should focus on improving our understanding of the molecular and biochemical changes at play in the degeneration and regeneration of injured peripheral nerves and the mechanisms by which neurotrophic factors effect change. Exogenous administration of pharmaceutical agents must also be explored further to optimize timing and dosage of agents released to maximize their therapeutic impact. Gene therapy and sophisticated nerve conduits present additional opportunities to overcome these challenges. Another highly promising avenue is the use of pluripotent stem cells as many of these cell types are capable of differentiating and producing neurotrophic factors themselves to curate a neuroprotective, regenerative milieu that meets the needs of the injured axons as they change over both time and distance. There may also be synergistic benefits to combining molecular therapies with other procedural modalities like intraoperative electrical stimulation. Ultimately, improvement of peripheral nerve regeneration after injury will likely require a synergistic combination of many of the above therapies to effectively address the complex nature of axonal regrowth and remyelination.

## Conflicts of Interest

No benefits in any form have been received or will be received related directly to this article.
